# Decoding contextual crosstalk: revealing distinct interactions between non-coding RNAs and unfolded protein response in breast cancer

**DOI:** 10.1186/s12935-024-03296-3

**Published:** 2024-03-11

**Authors:** Negin Karamali, Arshia Daraei, Arman Rostamlou, Roya Mahdavi, Zahra Akbari Jonoush, Nooshin Ghadiri, Zahra Mahmoudi, Amirhossein Mardi, Moslem Javidan, Sepideh Sohrabi, Behzad Baradaran

**Affiliations:** 1 Department of Immunology, School of Medicine, Tabriz University of Medical Sciences, Tabriz, Iran; 2 Immunology Research Center, Tabriz University of Medical Sciences, Tabriz, Iran; 3 Department of Immunology, School of Medicine, Kermanshah University of Medical Sciences, Kermanshah, Iran; 4 Department of Medical Biology, School of Medicine, University of EGE, Bornova, Izmir Turkey; 5 Student Research Committee, Ahvaz Jundishapur University of Medical Science, Ahvaz, Iran; 6 Department of Immunology, School of Medicine, Ahvaz Jundishapur University of Medical Sciences, Ahvaz, Iran; 7 Department of Immunology, School of Medicine, Shiraz University of Medical Sciences, Shiraz, Iran

**Keywords:** Breast cancer, Non-coding RNAs, ER stress, UPR, Therapy

## Abstract

Breast cancer is significantly influenced by endoplasmic reticulum (ER) stress, impacting both its initiation and progression. When cells experience an accumulation of misfolded or unfolded proteins, they activate the unfolded protein response (UPR) to restore cellular balance. In breast cancer, the UPR is frequently triggered due to challenging conditions within tumors. The UPR has a dual impact on breast cancer. On one hand, it can contribute to tumor growth by enhancing cell survival and resistance to programmed cell death in unfavorable environments. On the other hand, prolonged and severe ER stress can trigger cell death mechanisms, limiting tumor progression. Furthermore, ER stress has been linked to the regulation of non-coding RNAs (ncRNAs) in breast cancer cells. These ncRNAs, including microRNAs (miRNAs) and long non-coding RNAs (lncRNAs), play essential roles in cancer development by influencing gene expression and cellular processes. An improved understanding of how ER stress and ncRNAs interact in breast cancer can potentially lead to new treatment approaches. Modifying specific ncRNAs involved in the ER stress response might interfere with cancer cell survival and induce cell death. Additionally, focusing on UPR-associated proteins that interact with ncRNAs could offer novel therapeutic possibilities. Therefore, this review provides a concise overview of the interconnection between ER stress and ncRNAs in breast cancer, elucidating the nuanced effects of the UPR on cell fate and emphasizing the regulatory roles of ncRNAs in breast cancer progression.

## Introduction

Non-coding RNAs (ncRNAs) are RNA molecules that play crucial roles in various cellular processes. They are transcribed from DNA, similar to messenger RNAs (mRNAs), but instead of being translated into proteins, they function as RNA molecules. There are several classes of ncRNAs, each with distinct structures and functions. Some well-known classes include microRNAs (miRNAs), Long Non-coding RNAs (lncRNAs), Small Interfering RNAs (siRNAs), and circular RNAs (circRNAs). miRNAs are small ncRNAs that regulate gene expression by binding to target messenger RNAs, while lncRNAs are longer ncRNAs that regulate gene expression at various levels [1]. siRNAs are double-stranded ncRNAs that guide the silencing machinery to degrade complementary target RNAs or inhibit their translation. circRNAs form covalently closed circular structures and can act as miRNA sponges, sequestering miRNAs and modulating gene expression. The roles of ncRNAs in development, cancer, neurological disorders, and other diseases are actively investigated, with significant potential as diagnostic markers and therapeutic targets in various fields of medicine and biotechnology [2–4]. Evidence suggests that endoplasmic reticulum stress (ER stress) can affect the expression and activity of specific ncRNAs in breast cancer cells [5–7]. The ER is a vital cellular organelle that plays a crucial role in protein synthesis, folding, and maintaining cellular homeostasis. ER stress can lead to protein homeostasis disorder, resulting from factors like disturbances in calcium homeostasis, nutrient deprivation, oxidative stress, viral infection, or genetic mutations. The ER regulates proteostasis through processes like the UPR, the ubiquitin–proteasome system (UPS), and autophagy. The UPR increases the production of chaperone proteins, reduces protein synthesis, and activates ER-associated degradation (ERAD). When ER stress exceeds the cellular capacity, it can cause cellular dysfunction and contribute to diseases like neurodegenerative disorders, metabolic diseases, and cancer [8–10]. Studies have shown that UPR can modulate the expression of miRNAs involved in breast cancer progression. Dysregulation of certain miRNAs can impact cancer cell survival, proliferation, and migration [11]. Furthermore, some lncRNAs, which are regulators of UPR genes, can interact with UPR-associated proteins and affect the expression or activity of ER stress-related genes [12]. Attaining a comprehensive comprehension of the intricate interplay between ncRNAs and ER stress within the context of breast cancer holds the potential to revolutionize treatment strategies significantly. This knowledge possesses the capability to unveil novel therapeutic targets, fostering the development of personalized approaches grounded in distinctive molecular signatures. Enhancing our understanding of how ncRNAs specifically impact ER stress response pathways could enhance the efficacy of current treatments, sensitize cancer cells to standard therapies, and address resistance mechanisms. Additionally, the identification of diagnostic and prognostic biomarkers within the ncRNA-ER stress axis can inform treatment decisions and monitoring processes. Integrating ncRNA-targeted therapies with conventional methods may yield synergistic effects, and manipulating the tumor microenvironment shows promise for augmenting responses to immunotherapies. Ultimately, these further discoveries may play a crucial role in the long-term management of the disease, offering new avenues to sustain control and prevent relapse in breast cancer. The objective of this review is to explore the interaction between ncRNAs and ER stress response pathways in breast cancer cells.

## Role of non-coding RNAs in the pathogenesis of breast cancer

In the year 2020, breast cancer emerged as the most commonly diagnosed cancer on a globally, registering over 2.3 million new cases and resulting in 685,000 associated deaths. Notably, a majority of these cases were documented in transitioned countries, although a disproportionately high number of breast cancer-related deaths also occurred in transitioning countries. The terms “transitioning”, and “emerging” are used interchangeably to describe nations classified as low or medium Human Development Index (HDI). Conversely, “transitioned” is used to refer to those classified as high or very high HDI. Projections indicate a significant rise in the burden of breast cancer in the coming years, with estimations surpassing 3 million new cases and 1 million deaths by the year 2040. These projections raise serious concerns, emphasizing the critical need for the implementation of comprehensive and effective global policies aimed at combating the devastating consequences of breast cancer and mitigating its escalating impact [13]. The etiological underpinnings of breast cancer are inexorably intertwined with the dysregulation of ncRNAs, a phenomenon that has captured the attention of researchers in recent years [14]. ncRNAs, when their expression deviates from the normal levels, play a crucial role in regulating essential cellular processes such as cell proliferation, apoptosis, migration, and invasion, actively contributing to the initiation and progression of breast cancer [15, 16]. Extensive research efforts have been made to unravel the functional relevance of ncRNAs in the pathogenesis of breast cancer. In a 2019 study, researchers identified a significant overexpression of miR-21 in the breast in patient with breast cancer. Additionally, a reduction in the expression of programmed cell death protein 4 (PDCD4) was observed in these patients. The PDCD4 gene can inhibit tumor growth and promote cell death via the Sp1 transcription factor. The increased expression of miR-21 was found to accelerate mammary cell transformation and the growth of mammary tumors by inducing translational suppression of PDCD4 [17]. In 2023, Huang, Xie et al. identified elevated levels of miR-1260b and reduced mRNA expression of secretory coil domain-containing protein 134 (CCDC134) in human breast cancer tissue and a panel of human breast cancer cell lines. Silencing miR-1260b resulted in a decrease in the migration and invasion capabilities of breast cancer cells. Furthermore, the findings demonstrated that upregulating CCDC134 hindered the motility and invasion of breast cancer. These results suggest that miR-1260b functions as an oncogene in breast cancer, potentially promoting the motility and invasion of BRCA cells by suppressing the target gene CCDC134 and activating the MAPK signaling pathway. Additionally, it may play a role in suppressing immunological function and enhancing the immune system [18]. Furthermore, lncRNAs play a vital role in promoting the growth of breast cancer. A study in 2018 revealed a novel connection between the lncRNA LINC00538 (YIYA) and cancer metabolism in breast cancer. In a cell cycle-independent manner, YIYA interacts with cytosolic cyclin-dependent CDK6 kinase and controls CDK6-dependent phosphorylation of fructose bisphosphatase PFK2 (PFKFB3). These events increase the catalysis of glucose 6-phosphate to fructose-2,6-bisphosphate/fructose-1,6-bisphosphate in breast cancer cells. Silencing YIYA or CDK6 by CRISPR/Cas9-mediated deletion can disrupt glycolysis and tumor development in vivo. Approximately 40% of clinical breast cancer samples showed the presence of YIYA, which is associated with poor survival outcomes and CDK6 expression. These findings underscore the functional significance of lncRNAs in metabolic reprogramming [19]. A recent study highlighted that lncSNHG5 is significantly expressed in breast cancer-associated fibroblasts (CAFs) and plays a crucial role in the formation of the pre-metastatic niche. This is achieved by enhancing angiogenesis and vascular leakage through the regulation of Zinc finger protein 281 (ZNF281) in CAFs. The interaction between lncSNHG5 and the IGF2BP2 m6A reader contributes to the increased stability of ZNF281 mRNA. In endothelial cells, ZNF281 is responsible for the transcriptional control of CCL2 and CCL5 expression, as well as the activation of MAPK p38 signaling. The overexpression of CCL2 and CCL5 is associated with tumor metastasis and a poor prognosis in patients. Ultimately, the study findings underscore the critical role of the lncSNHG5-ZNF281-CCL2/CCL5 signaling axis in promoting the establishment of the pre-metastatic niche, thereby facilitating breast cancer metastasis [20]. circRNAs, akin to miRNAs and lncRNAs, play an integral role in the pathophysiology of breast cancer. Research has demonstrated significant expression of circSKA3 in breast cancer tissues and cells, directly correlating with the invasive capacity of breast cancer cells through the formation of invadopodia. The binding partners of circSKA3 in invadopodia derived from tumors were identified as Tks5 (tyrosine kinase substrate with 5 SH3 domains) and integrinb1. Furthermore, abnormal expression of circSKA3 was found to enhance tumor invasiveness both in vitro and in vivo [21]. In 2022, Qi et al. uncovered the involvement of circ_RPPH1 and Rho GTPase activator protein 1 (ARHGAP1), coupled with the decreased expression of miR-542-3p, in breast cancer (BC) tissues of cancer patients. The knockdown of circ_RPPH1 or the introduction of miR-542-3p resulted in a reduction in breast cancer cell growth and metastasis, accompanied by the induction of apoptosis. circ_RPPH1 acts by inhibiting miR-542-3p, which, in turn, regulates ARHGAP1 expression, thereby influencing the progression of breast cancer. Additionally, the downregulation of circ_RPPH1 was observed to inhibit tumor growth in vivo [22]. In conclusion, the findings from these studies highlight the intricate involvement of ncRNAs in essential cellular processes like proliferation, apoptosis, migration, and invasion, playing an active role in the onset and advancement of breast cancer. By comprehending the impact of these ncRNAs on diverse pathways, valuable insights can be gained regarding potential targets for therapeutic interventions and diagnostic markers in breast cancer.

## Role of the endoplasmic reticulum stress in the pathogenesis of breast cancer

**Fig. 1:**
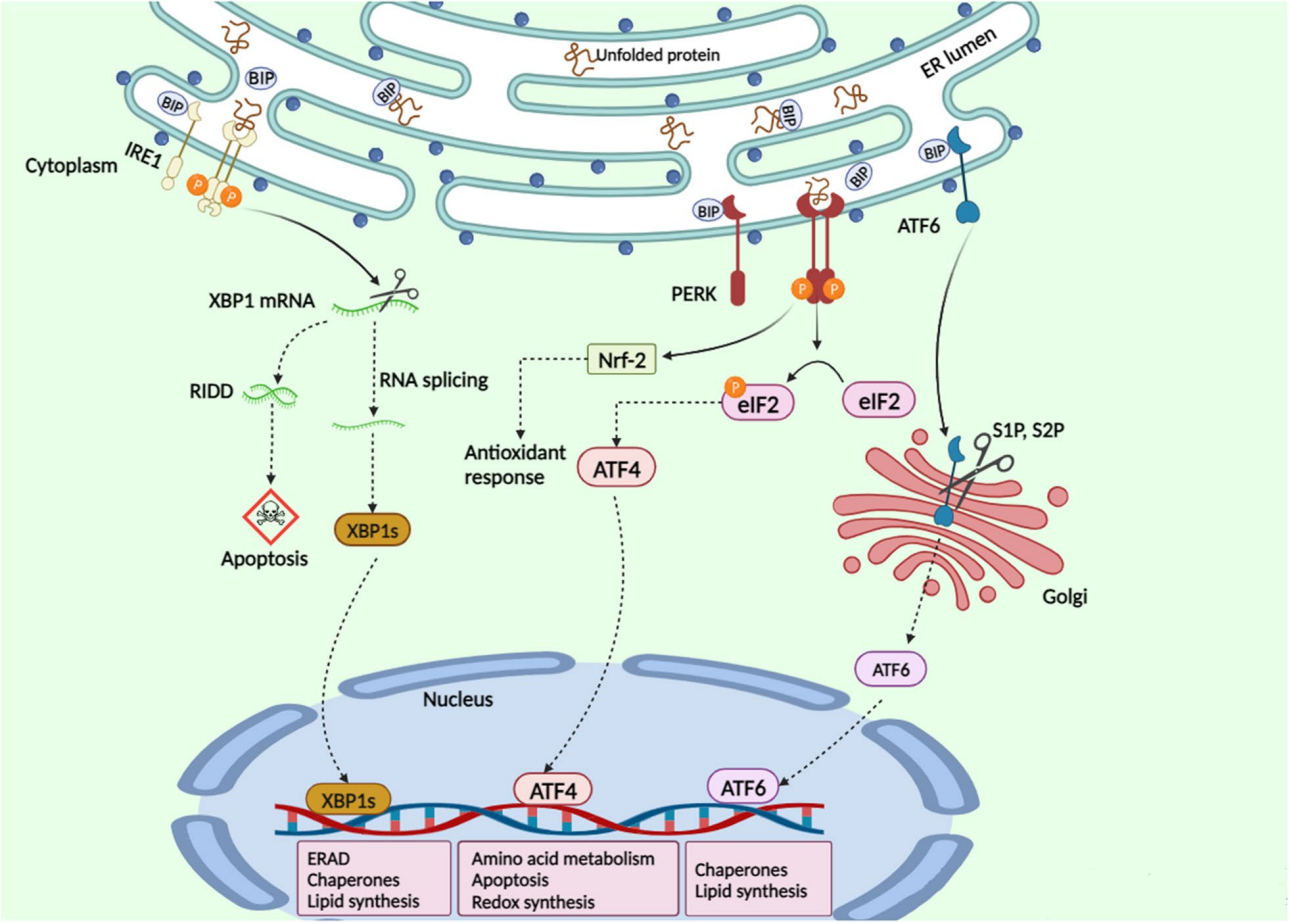
Endoplasmic reticulum (ER) stress is the result of an imbalance or disturbance in the normal functioning of the ER, a cellular organelle involved in protein production and folding. An excess of misfolded proteins, a calcium imbalance, a viral infection, or dietary deficiency can all contribute to this. When ER stress occurs, a cellular signaling cascade called the unfolded protein response (UPR) is triggered. It aims to reduce stress and return to ER equilibrium. IRE1, PERK, and ATF6 are the three primary branches of the UPR that are mediated by distinct ER transmembrane proteins. IRE1 pathway: IRE1 splices and processes XBP1 mRNA to produce a transcription factor that activates genes related to ER-associated degradation and protein folding. PERK pathway: eIF2α is phosphorylated by PERK, which causes an overall decrease in protein synthesis. It preferentially stimulates the translation of ATF4, which controls the expression of genes related to the metabolism of amino acids, apoptosis, and antioxidant responses. ATF6 pathway: after being delivered to the Golgi apparatus, ATF6 cleaves and releases a fragment of a transcription factor. After translocating to the nucleus, this fragment activates genes related to lipid metabolism, ER-associated degradation, and protein folding. Stress reduction and ER function restoration are the primary objectives of the UPR. Normality is restored to cellular activities upon resolution of the stress. On the other hand, apoptotic mechanisms may cause cell death in response to extended or severe ER stress. Created by https://www.biorender.com/

The ER is a cellular organelle responsible for protein synthesis and homeostasis. ER stress occurs when the ER struggles to fold proteins correctly, resulting in the buildup of misfolded proteins. To counter this, the UPR is activated as a cellular mechanism to restore equilibrium (Fig. 1). Under normal conditions, cells employ a process known as the UPR to manage stress within the ER. The UPR mechanism involves several strategies to address ER stress effectively. Firstly, it promotes the synthesis of chaperone proteins, which aid in the proper folding of newly synthesized proteins, ensuring their functionality. Additionally, the UPR reduces the overall production of proteins, alleviating the burden on the ER. This reduction in protein synthesis helps restore ER homeostasis. Moreover, the UPR activates a process called ERAD. ERAD is responsible for identifying and eliminating proteins that fail to fold correctly, preventing the accumulation of misfolded or dysfunctional proteins within the ER. Overall, the UPR acts as a protective mechanism to maintain ER function and cellular homeostasis in response to ER stress [8, 9]. The UPR is regulated by three primary sensors: inositol-requiring enzyme 1 (IRE1), protein RNA-activated kinase (PKR)-like ER kinase (PERK), and activating transcription factor 6 (ATF6). Its primary role is to restore normal functioning of the endoplasmic reticulum (ER) by repairing or degrading proteins. However, if the stress persists, it can trigger cell death, including apoptosis, and contribute to the development of diseases such as cancer. ER stress-induced apoptosis serves as a crucial cellular mechanism for eliminating damaged cells, maintaining protein balance, and ensuring proper tissue development. Understanding and effectively managing this process are essential in various scenarios. Targeting the UPR, particularly the aspect related to ER stress-induced apoptosis, holds potential as a therapeutic strategy, particularly in conditions like cancer [10, 23]. ER stress can occur in cancer cells because of their rapid growth, insufficient nutrients, and improper protein folding. This susceptibility can be exploited for targeted therapy, aiming to kill cancer cells while sparing healthy ones. Proteasome inhibitors and combination therapies disrupting protein folding and ER function are among the medications and therapeutic approaches designed to induce ER stress and promote apoptosis in cancer cells [24]. Evidence from studies suggests that the upregulation of UPR components contributes to the proliferation, progression, and chemotherapy resistance in breast cancer cells [25]. The activation of PERK-ATF4 is pivotal for the advancement and rapid growth of breast cancer cells, as supported by experimental results obtained both in vitro and in vivo by Feng in 2017 [26]. Dysregulation of the PERK arm leads to the modification of signaling pathways, establishing an adaptive mechanism that enables cell survival in challenging circumstances. This suggests that disruptions in the normal functioning of the PERK pathway prompt cells to adapt and find alternative means to ensure survival in unfavorable environments or circumstances. Understanding these adaptive mechanisms is crucial, especially in diseases like cancer, where these changes may contribute to the advancement and resilience of cancer cells [27, 28]. Recent studies have shown that inhibiting the PERK pathway can increase the susceptibility of breast cancer cells to apoptosis, indicating its potential as a treatment strategy. However, it is important to note that separate research has indicated that ATF4 can independently influence the regulation of autophagy, irrespective of PERK. This suggests that while inhibiting PERK may promote apoptosis, the involvement of ATF4 and autophagy regulation adds complexity, requiring a comprehensive understanding of the overall cellular response in breast cancer. The interplay between these pathways highlights the intricate nature of cellular processes and the challenges in developing targeted therapies [29]. The activation of PERK-ATF4 in autophagy has the potential to tilt the balance from pro-survival signals to pro-death signals, disrupting the equilibrium of apoptosis [30]. Other components of the UPR include chaperones like GRP78 (BiP) and GRP94 (gp96). GRP78, a crucial molecular chaperone, aids in the folding, assembly, and secretion of proteins within the ER, playing a vital role in the UPR. Another ER-resident chaperone, GRP94, interacts specifically with secretory and transmembrane proteins. Notably, GRP94’s immunogenic characteristics have led to its exploration in immunotherapy for diseases such as cancer. Overexpression of both GRP78 and GRP94 is often observed in malignancies, including breast cancer, highlighting their significance in disease development. Given their role in maintaining protein homeostasis and their association with diseases characterized by protein misfolding, targeting these chaperones presents a promising therapeutic approach [31, 32]. In the absence of GRP78, which is an ER chaperone protein, CHOP initiates a notable impact on impeding the formation of malignant tumors. This is achieved by triggering cell death in precancerous cells, and the mechanism involves the downstream modulation of the PERK signaling pathway. The disruption of the GRP78 gene, leading to the activation of CHOP and subsequent modulation of PERK signaling, acts as a deterrent to the development of cancerous growths, emphasizing the intricate relationship between ER stress response, cell survival, and tumorigenesis [33]. The PERK signaling pathway exhibits a dual role, capable of both promoting and suppressing tumor progression. In the context of breast tumors, this pathway holds the potential to impede tumor progression. However, it is crucial to note that prolonged activation of the PERK pathway may lead to the induction of breast cancer by fostering genomic instability [34]. Abundant research has accumulated compelling evidence affirming that therapeutic interventions targeting PERK, GRP78, and IRE1/XBP1 have substantial potential to enhance the effectiveness of breast cancer treatment. The utilization of inhibitors directed at these proteins, combined with drugs inducing resistance through the UPR, has shown promise in reducing the growth of breast tumors in vivo [35]. ER stress exerts effects beyond the proteins directly involved in its pathway. A 2014 study demonstrated that inhibiting ER stress-induced heparinase expression in breast cancer cells yields positive outcomes, reducing tumor invasion and metastasis. This inhibition also upregulates CCL5 expression, subsequently reducing transmigration. Notably, CCL5 is recognized for inducing tumor angiogenesis, impacting tumor cell motility, invasion, and metastasis [35]. Certainly, ER stress exhibits a dual role in breast cancer progression. Firstly, it can hinder breast cancer development by triggering cell death pathways during prolonged stress conditions. Conversely, ER stress sustains the progression of breast cancer by fostering adaptive mechanisms that aid the survival of cancer cells. This dual nature emphasizes the significance of targeting ER stress in therapeutic interventions for breast cancer treatment. Approaches focused on modulating ER stress-induced pathways, including the inhibition of PERK or other UPR components, show promise in improving treatment effectiveness and potentially restraining breast cancer metastasis.

## Non-coding RNAs and ER stress

### Non-coding RNAs involved in the folding and modification of proteins

Proteins must be accurately folded into precise, three-dimensional forms to function effectively, which is an essential biological activity. The ER is a cellular compartment dedicated to the process of protein folding. As noted, the failure of proteins to fold correctly leads to ER stress. Calreticulin (CALR), BIP/GRP78, and protein disulfide isomerases (PDIs) are indeed vital chaperone proteins located in the ER lumen. They play critical roles in preserving protein homeostasis, ensuring the proper folding, and maintaining the quality control of newly synthesized proteins [36]. To investigate the role of miR-455 in the protective effect of H2S on lung epithelial cells against CoCl2-induced apoptosis through the modulation of ER stress-related genes, experiments were performed using human lung epithelial cells (BEAS-2B). These experiments included exposing the cells to hypoxic injury, both with and without prior H2S preconditioning. Researchers identified that CALR, GRP78, CHOP, and Caspase-12 protein expression were all downregulated by the miR-455 mimic, whereas their expression was increased by the miR-455 inhibitor [37]. In another study, it has been shown that miR-663a may act as a CALR regulator in patients with rectal cancer [38]. GRP78 and CALR, two ER stress indicators, are downregulated by miR-124 in angiotensin II (AngII)-induced myocardial hypertrophy. This suggests that the observed inhibition of cardiac hypertrophy resulting from miR-124 downregulation may be attributed to the reduction of ER stress [39]. Based on the data currently available, miR-455 has binding sites in CALR mRNA, and microRNA sequencing discovered that miR-455 is downregulated in the hearts of mice with constitutively active ATF6. Additionally, the induction of the UPR with tunicamycin or the overexpression of constitutively active ATF6 in neonatal rat ventricular myocytes (NRVMs) results in reduced levels of miR-455 and increased expression of CALR. Furthermore, CALR abundance is reduced as a result of overexpressing pre-miR-455, leading to elevated levels of miR-455. Conversely, CALR abundance increases when miR-455 antagonistic oligonucleotides are transfected, thereby reducing their levels. This indicates that, by decreasing the levels of miR-455, ER stress at least partially contributes to the activation of CALR [40, 41]. Soft tissue sarcomas, known as liposarcomas, are characterized by poor adipocyte development, especially in the dedifferentiated subtype. The dysregulation of CALR, possibly regulated by miR-1257, may contribute to the inhibition of adipocyte development, potentially explaining the dedifferentiated phenotype observed in liposarcomas [42]. Regulation of BIP is essential for maintaining ER Ca2+ homeostasis and protein folding because it is a master regulator of the UPR, which is vital for ER Ca2+ storage [43]. Heat shock 70 kDa protein 5 (HSPA5) encodes the protein GRP78. Recent research has revealed a significant presence of GRP78 on the cell surface of cancer cells. Consequently, GRP78 has emerged as a crucial regulator of signaling pathways associated with tumor cell viability. This discovery has prompted the investigation of therapeutic strategies targeting cell surface GRP78 for cancer treatment [44]. In laboratory experiments conducted both in vitro and in vivo, the introduction of a miR-181a mimic has been shown to decrease GRP78 protein expression, indicating negative regulation by miR-181a. Conversely, using a miR-181a inhibitor or antagomir results in an increase in GRP78 protein expression, implying that inhibiting miR-181a allows for higher levels of GRP78 protein production [45]. miR-30d, miR-181a, and miR-199a-5p collaborate to control GRP78 levels by destabilizing GRP78 mRNA, resulting in reduced levels at both the mRNA and protein levels. This cooperative action is a common phenomenon in gene regulation, where multiple miRNAs fine-tune the expression of target genes. Further research is needed to comprehend the precise mechanisms underlying the destabilization by these miRNAs and the functional significance of this regulatory network in various biological processes and disease conditions [46]. According to reports, overexpressing lncRNA RP11-115N4.1 dramatically reduces K562 cell proliferation and modifies the immunological response by triggering HSP70 production by binding to HNRNPH3 [47]. The results strongly suggest that the lncRNA Hotair functions as a positive regulator in laryngeal squamous cell carcinoma by influencing the stability of GRP78 through posttranscriptional modifications, coordinated by the regulation of hsa-miR-30a-5p. Moreover, Hotair facilitates immune evasion by upregulating the expression of PD-L1 through the hsa-miR-30a-5p/GRP78 pathway [48]. Overexpression of miR-204 results in a decrease in the activation or expression of specific genes responsive to ER stress, namely GRP78, GRP94, and CHOP. This reduction in gene induction is associated with the phenotypic characteristics typically observed in senescent cells [49]. There is a strong association between HOTAIR and HSPA1A, a member of the heat shock protein family A (Hsp70), in breast cancer (BRCA) tissues. HOTAIR was found to elevate HSPA1A levels in BRCA cells after radiation exposure, affecting both mRNA and protein levels. miR-449b-5p, which normally inhibits HSPA1A production by binding to the 3′-UTR region of HSPA1A mRNA, is responsible for this action [50]. ER stress in the cardiovascular system is induced by reduced expression of miR-30 family miRNAs, leading to increased production of GRP78. This disruption in the regulatory activity of miR-30 family miRNAs hinders the normal physiological response to ER stress, impacting the balance of protein folding and processing. Ultimately, this imbalance may contribute to the development or progression of cardiovascular disorders [51]. Another study showed that NEAT1 expression is positively correlated with GRP78, but there is no further evidence of their interaction [52]. Another factor in protein folding is PDIs, which are regulated by microRNAs. PDIs, a class of oxidoreductases primarily located in the ER, play crucial roles in protein folding and maturation by catalyzing disulfide bond formation, isomerization, and reduction of nascent proteins [53]. Protein disulfide isomerase family A member 6 (PDIA6), also known as P5, is an enzyme belonging to the PDI family. PDIA6 plays a critical role in catalyzing protein folding processes, displaying both isomerase and chaperone activities. Interestingly, PDIA6 is downregulated during cellular senescence in bone marrow-derived mesenchymal stem cells (BMSCs) [54]. In 2023, Huang et al. demonstrated that in H_2_O_2_-treated human foreskin fibroblasts (HFF), the expression of PDIA6 is reduced compared to the control group. Interestingly, inhibition of miR-181a alleviates oxidative stress induced by H_2_O_2_ and reduces cellular senescence in HFF. Moreover, when PDIA6 is knocked down in combination with the downregulation of miR-181a, the inhibitory effects on cellular senescence and oxidative stress are reversed. This suggests that silencing miR-181a can mitigate H_2_O_2_-induced cellular senescence and oxidative stress by targeting PDIA6. Additionally, the findings suggest that PDIA6 may play a protective role in HFF, shielding them from cellular senescence induced by H_2_O_2_ [55]. The UPR pathway responds to disturbances in calcium homeostasis and is regulated by the ER oxidoreductase PDIA6 and miR-322. PDIA6 interacts with IRE1α, enhancing its activity by increasing phosphorylation and splicing of XBP1 mRNA. PDIA6 has minimal effects on other pathways involved in the cellular response to ER stress. Depletion of ER Ca2+ levels result in decreased levels of miR-322, leading to increased stability of PDIA6 mRNA and enhanced IRE1α activity during the ER stress response [56]. Regardless of the factors involved, ncRNAs contribute to the intricate regulatory networks governing protein folding and modification. The complexity of these processes and the importance of ncRNAs in maintaining protein homeostasis and cellular function are underscored by their involvement in multiple pathways of action. Understanding the role of ncRNAs in protein homeostasis involves examining their functions in related pathways, particularly those involved in cellular stress responses such as the UPR. Here’s a closer look at how ncRNAs function in these pathways.

### Non-coding RNAs in ER stress

**Fig. 2:**
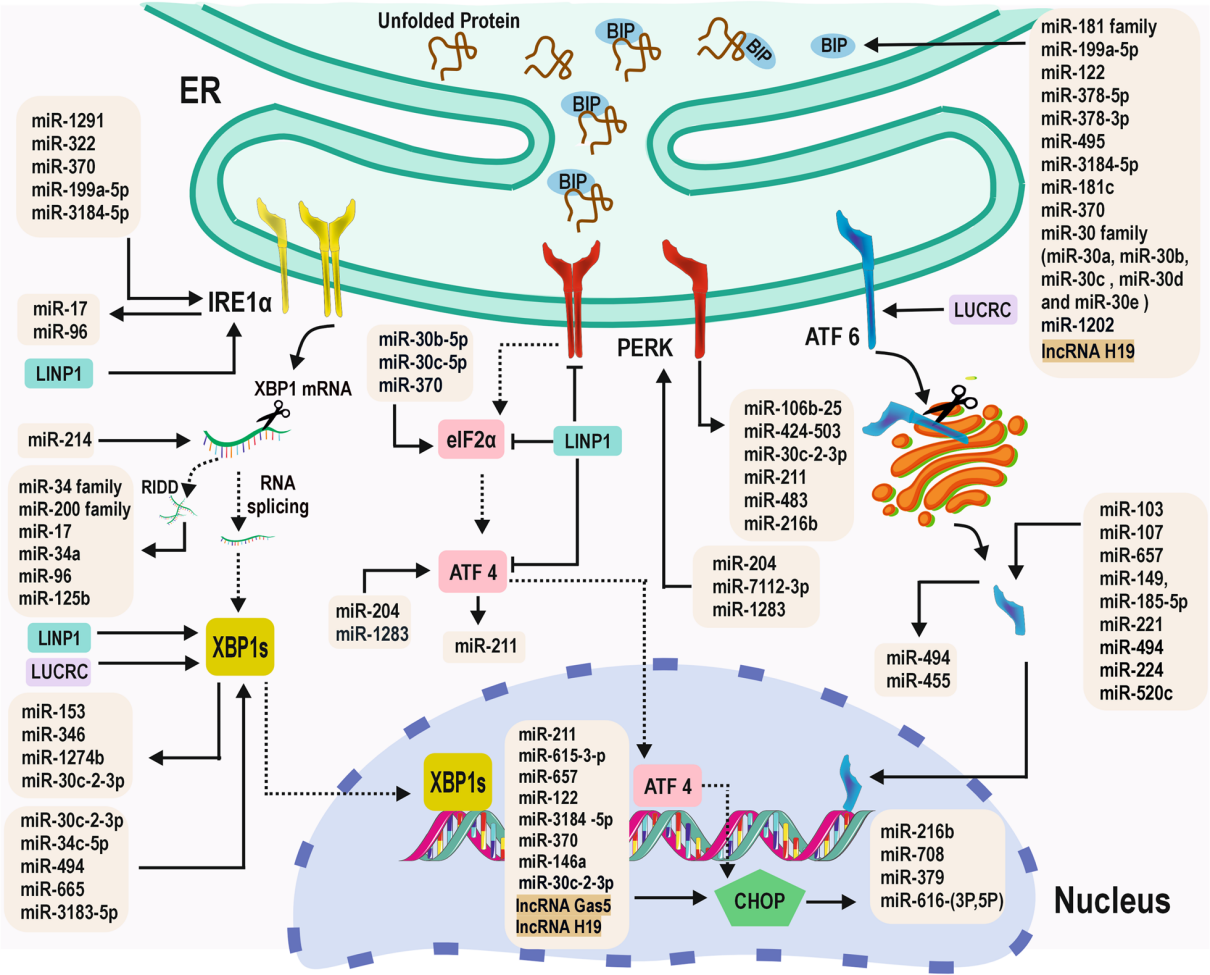
A model to depict the interaction between non-coding RNAs and ER stress

Observations from mammalian cells suggest that ncRNAs play a role in determining ER stress, and, reciprocally, ER stress also controls ncRNA expression [19] (Fig. 2). Numerous studies have explored the impact of ncRNAs on ER stress in various disorders, and we have previously provided an overview of ncRNAs involved in protein folding. XBP1 regulates miR-346 and miR-153 during ER stress, while certain miRNAs such as miR-34c, miR-665, and miR-30c directly target XBP1 mRNA [57]. The study indicates that miRNAs play a role in hindering the transition from cellular survival to programmed cell death. Predominantly, miRNAs implicated in cell death induced by ER stress are located within the PERK–eIF2α–CHOP signaling pathway. miRNAs such as miR-17, miR-34a, miR-96, and miR-125b are crucial in orchestrating the transition of the PERK–eIF2α–CHOP axis towards cell death in response to ER stress. This underscores the significance of these miRNAs in the context of cancer-related cell death [58]. Several other miRNAs, including miR-24, miR-29, miR-195, miR-221, miR-222, miR-346, and miR-665, have been linked to cell death induced by ER stress through their targeting of various factors [59–64]. miR-410 plays a regulatory role in ER stress, facilitating tumor progression by suppressing cellular migration and invasion in breast cancer cells. This effect is achieved through the specific modulation of gene expression, including CHOP, GRP94, Bip, and p-PERK [65, 66]. miR-30b-5p and miR-30c-5p have been observed to target the eIF2α protein, promoting cell survival in hepatocellular carcinoma (HCC) and breast cancer when treated with PS-341 [67]. PS-341 is known to impede ERAD and result in the accumulation of misfolded proteins [68]. The expression of PD-L1 in macrophages, promoting immune evasion of cancer cells, is activated by cancer-derived exosomal microRNAs (exo-miRs) induced by ER stress. This discovery establishes a novel connection between cancer and ER stress, elucidating a previously unknown mechanism [69]. Other ncRNA types, such as snoRNAs and siRNAs, may play a role in regulating ER stress. However, the specific roles and mechanisms of action of these ncRNAs in ER stress are still being explored. In general, ncRNAs are crucial for controlling ER stress and preserving protein homeostasis. They play a vital role in the adaptive response to ER stress, preventing cellular malfunction and disease by regulating the expression of critical genes involved in ER stress signaling pathways.

### Non-coding RNAs as biomarkers in UPR

ncRNA molecules has become crucial in pinpointing biomarkers indicative of disease development and progression. The field of RNA therapeutics is undergoing a transformative phase with notable progress in oligonucleotide techniques and pharmacological discoveries. Targeting RNA structures and RNA–protein interactions with small molecules presents a promising avenue for developing the next generation of drugs. These advancements enable precise modulation of gene expression, offering potential therapeutic interventions at the genetic level [70]. Recently emerging findings highlight the pivotal roles of ncRNAs, including miRNAs and lncRNAs, in regulating UPR signaling pathways and shaping cellular responses to ER stress. These ncRNAs act as highly sensitive indicators of UPR irregularities, providing insights into the intricate molecular mechanisms of stress-related conditions like neurodegenerative disorders and specific cancers. Investigating the distinct signatures of ncRNAs associated with UPR activation or malfunction allows researchers to establish these molecules as reliable biomarkers for monitoring cellular stress and tracking disease progression. Effectively communicating the significance of ncRNAs in the UPR framework not only enhances our understanding of cellular stress responses but also presents significant opportunities for advancing diagnostic and therapeutic approaches in disorders linked to UPR dysregulation. Table 1 compiles various ncRNAs that undergo alterations when subjected to ER stress induction and UPR activation, providing insights into their functional mechanisms.

**Table 1:** Non-coding RNAs involved in endoplasmic reticulum stress pathway regulation

Non-coding RNAs	Mechanisms	Subjects	Study type	References
LINP1	LINP1 negatively regulates UPR pathways, particularly through interaction with eIF2α, preventing excessive UPR activation and contributing to cSCC development suppression	HSC-1, A43, and HaCaT cells Mice	In vitro and in vivo	[132]
LUCRC	Knockdown of LUCRC in HCT116 cells results in differential regulation of genes. LUCRC positively regulates BIP expression, and its depletion led to altered splicing of XBP1 and reduced ATF6 processing in response to ER stress	HCT116 cells Human Mice	In vitro and in vivo	[133]
GAS5	In ARPE-19 cells exposed to high glucose (HG), upregulation of GAS5 results in a decrease in total protein expression levels of ATF4 and CHOP. Additionally, there is a reduction in the relative protein phosphorylation ratio of p-PERK/PERK and p-eIF2α/eIF2α compared to control groups	ARPE-19 cells	In vitro	[134]
H19	H19 is associated with a reduction in the expression of various proteins related to ER stress, including p-PERK, p-IRE1α, ATF6, CHOP, cleaved caspase-3, cleaved caspase-9, cleaved caspase-12, and BAX in cardiac tissues	HL-1 cells Mice	In vitro and in vivo	[135]
MIR503HG	LncRNA MIR503HG has been found to act as a sponge for miR-224-5p, leading to the upregulation of TUSC3. This, in turn, results in the suppression of the ATF6 branch of the UPR and the development of gastric cancer	SGC7901 and BGC-823 Mice Human	In vitro and in vivo	
miR-1291	In silico predictions and experimental validation suggest that miR-1291 represses the expression of IRE1α mRNA	HuH7 cells	In vitro	[136]
miR-424	miR-424 appears to be intricately involved in the regulation of UPR by influencing the expression of ATF6, PERK signaling, and RIDD, with its downregulation being a part of the response to ER stress, mediated by PERK	MEFs, H9c2, and HEK 293T cells Mice	In vitro and in vivo	[137]
miR-322	The ER oxidoreductase PDIA6 and miR-322 are identified as key regulators of IRE1α activity. The reduction in ER Ca2+ levels and activation of store-operated Ca2+ entry led to decreased miR-322 abundance, subsequently stabilizing PDIA6 mRNA and amplifying IRE1α activity during ER stress	embryonic fibroblasts and COS-1 cells Mice	In vitro and in vivo	[56]
miR-199a-5p	The research suggests that the protective impact of HUVEC-derived miR-199a-5p on neural cells occurs through exosome-mediated transfer, leading to the inhibition of ER stress-induced apoptosis and inflammation by targeting BIP	HUVECs, SH-SY5Y cells	In vitro	[138]
miR-3184-5p	XBP1 has identified as a target gene for miR-3184-5p, and downstream molecular effects implicate the regulation of CD44, cyclin D1, MMP2, p65, p-AKT, p-STAT3, GRP78, IRE1, p-JNK, CHOP, caspase-12, and BCL-2	HGC-27 cells Human	In vitro	[139]
miR-30c-2-3p	The research demonstrates that miR-30c-2-3p inhibits the expression of XBP1, leading to a decrease in the ER folding capacity and an intensification of ER stress, as evidenced by Thioflavin T staining. The study further reveals that miR-30c-2-3p up-regulates pro-apoptotic proteins CHOP and BIM while down-regulating the ER stress response protein BIP/GRP78	OVCAR3 (C430) and SKOV3 (C209) cells	In vitro	[140]
miR-34c	Functional experiments demonstrate that overexpressing miR-34c or suppressing HMGB1 leads to inhibited cell proliferation, increased apoptosis, and induction of ER stress in NSCLC cells	MRC-5, A549, H460, H157, H1299, and H23 cells	In vitro	[141]
miR-494	The finding indicates that miR-494 negatively regulates ER stress in HUVECs. When miR-494 levels are increased (miR-494 mimic), there is a reduction in the expression of ER stress-responsive genes and proteins. Conversely, inhibiting miR-494 (miR-494 inhibitor) leads to an increase in the expression of ER stress-responsive genes	HUVECs cells	In vitro	[142]
miR-665	The data suggests that miR-665 targets the ER stress components XBP1 and ORMDL3. The predicted target sequence in the 3′-UTRs of these genes, along with experimental evidence supports the idea that miR-665 has a regulatory role in modulating the expression of XBP1 and ORMDL3 in the context of ER stress	Human	In vitro	[60]
miR-204	miR-204 has regulatory effects on ER stress markers in HTM cell lines. Without tunicamycin treatment, miR-204 alters the expression of GRP78/BIP and CHOP/DDIT3. Furthermore, in the presence of tunicamycin, miR-204 significantly inhibits the induction of ER stress markers (GRP94, GRP78/BIP, and CHOP/DDIT3)	HTM cells	In vitro	[49, 143]
miR-1283	The inhibition of miR-1283 appears to play a role in promoting ER stress through the regulation of the PERK/ATF4 pathway	CRL-1730 cells Mice	In vitro and in vivo	[144]
miR-7112-3p	miR-7112-3p appears to play a crucial role in regulating the PERK-ATF4-CHOP-caspase 3/8 signaling pathway	CX-1 cells	In vitro	[145]
miR-211	Functional experiments involving antagomir and miR-211 expression have revealed a relationship between miR-211 expression and CHOP accumulation following ER stress. miR-211 has shown to regulate CHOP at both the protein and mRNA levels	NIH 3T3 cells Mice Human	In vitro	[146]
miR-615-3p	miR-615-3p overexpression results in a significant decrease in CHOP protein levels under conditions of palmitate and tunicamycin treatment in both IRE-WT and Hepa 1–6 cells	Hepatoma cell line	In vitro	[147]
miR-181	Computational predictions suggested that miR-181 could target the 3′UTRs of three HSP70 family members. Experimental validation revealed a specific interaction between miR-181 and the 3′UTR of GRP78. This suggests that miR-181 may directly regulate GRP78	Primary astrocyte	In vitro	[45]
miR-378	The provided experimental data suggests an intricate relationship between miR-378 and XBP1 during ER stress. The study employed MCF7 cells and observed that miR-378 undergoes downregulation in response to ER stress induced by Brefeldin A	MCF7, MDA-MB-231, and ZR-75-1	In vitro	[148]
miR-199a-5p and miR-495	miR-199a-5p and miR-495 are suggested to interact with the 3′-UTR of GRP78, indicating a direct regulatory relationship	A459, QU-DB, HEK293T Human	In vitro	[149]
miR-1202	miR-1202-dependent inhibition of Rab1A is proposed to activate ER stress. Expression of GRP78, a marker of ER stress, significantly increases in cells transfected with miR-1202 vector. The protein expressions of Rab1A, Bcl-2, and Bax are altered in response to miR-1202, suggesting a potential link between miR-1202, Rab1A, ER stress, and apoptosis in glioma cells	U87, U251, U373, A172, and LN229 Human	In vitro	[150]
miR-103/107	The findings suggest that miR-103/107 plays a role in promoting ER stress-induced apoptosis in preadipocytes. The downregulation of miR-103/107 led to a reduction in ER stress markers, pro-apoptotic genes, and caspase-3 activity, indicating a protective effect against apoptosis in preadipocytes	Primary preadipocyte	In vitro	[151]
miR-149	In non-alcoholic fatty liver disease (NAFLD) mice, there is a notable increase in mRNA and protein expressions of GRP94 and Akt, indicating activation of the ATF6 pathway and ER stress. Transfection of miR-149 mimics into NAFLD mice led to a significant decrease in the expressions of GRP94 and Akt. This suggests that miR-149 inhibits the ATF6 signaling pathway and the expressions of its downstream proteins	Mice	In vitro and in vivo	[152]
miR-185	The overexpression of miR-185 attenuates ER stress-induced apoptosis in cardiomyocytes. This conclusion is supported by a reduction in the percentage of TUNEL-positive cardiomyocytes and changes in the protein levels of CHOP and cleaved-caspase 3 in response to increasing concentrations of miR-185 mimic	Cardiomyocytes	In silico and in vitro	[153]
miR-221/222	Downregulation of miR-221/222 protects HCC cells against ER stress-induced apoptosis. miR-221/222 mimics sensitize cells to apoptosis, while miR-221/222 inhibitors attenuate apoptosis, indicating a regulatory role for miR-221/222 in the apoptotic response to ER stress in HCC cells	HepG2 and SMMC-7721	In vitro	[63]
miR-520a	In Raji cells, miR-520a has found to significantly inhibit the expression of GRP78, GADD, p-PERK, and eIF2α. When Raji cells are treated with inhibitors of miR-520a, there is an observed increase in the expression levels of GRP78, GADD, p-PERK, and eIF2α	Raji cells	In vitro	[154]

## Non-coding RNAs involved in ER stress in breast cancer

As mentioned, the UPR is initially activated in response to ER stress as a protective mechanism to safeguard cells from the adverse effects of misfolded proteins within the ER. It serves as a cellular strategy to restore normal homeostasis. However, if the UPR fails to restore the cell’s equilibrium, it can ultimately trigger cell death [71]. Therefore, it seems logical to consider the UPR as a potential tumor-suppressive system. Nevertheless, the role of ER stress and the UPR in cancer remains a subject of intense debate due to numerous pieces of evidence suggesting that, in ER-stressed cancer cells, the UPR may promote tumor development [72]. Also, some specific ncRNAs can influence the UPR signaling pathway; conversely, UPR components can boost the production of certain ncRNAs. This connection is particularly relevant in cancer studies, offering potential insights for effective tumor control strategies [6, 73] (Table 2). In 2019, Zhang et al. demonstrated that overexpression of the lncRNA MEG3, also known as GTL2, FP504, or LINC00023, significantly inhibits breast cancer cell growth both in vitro and in vivo by increasing apoptosis in breast cancer cells. MEG3 was found to enhance the expression of proteins associated with the ER stress response, including GRP78, IRE1, PERK, and ATF6. Additionally, MEG3 increased the levels of pro-apoptotic proteins, namely CHOP and caspase-3 [7]. This suggests that ER stress-triggered apoptosis mediated by MEG3 may be a consistent phenomenon observed across different cancer types [74, 75]. In another study, the impact of inducing ER stress on the expression of lncRNAs is observed. ER stress induction, through the activation of the GRP78/OCT4/lncRNA MIAT/AKT pathway, is shown to lead to drug resistance in breast cancer cells. The commonly used chemotherapy drug, 5-fluorouracil (5-FU), triggers ER stress, resulting in the upregulation of key ER stress proteins and the activation of the regulatory pathway mentioned above. This, in turn, promotes the proliferation of breast cancer cells, supporting tumor progression [76]. As highlighted in this review, XBP1s serve as a crucial transcription factor regulating various genes related to ER homeostasis, cell viability, angiogenesis, metastasis, and drug resistance. This factor is generated through the activation of the IRE1 pathway, leading to the excision of a 26-nucleotide intronic segment. The splicing rate of XBP1 (XBP1 spliced/unspliced) is significantly increased in malignant breast tissue compared to non-malignant tissue. Notably, the upregulation of lncRNAs NORAD, NEAT1, and LINC00299 is linked to an elevation in the splicing rate of XBP1. Conversely, the expression of the lncRNA CASC2 exhibits a negative correlation, indicating that an increase in CASC2 gene expression is associated with a reduction in the splicing rate of XBP1 [77]. Furthermore, induced ER stress in breast tumor tissue appears to significantly influence cancer progression through its impact on miRNA expression. This induction leads to the enhanced exosomal expression and secretion of miR-27a-3p, consequently promoting increased immune evasion in breast cancer. MiR-27a-3p achieves this effect by upregulating PD-L1 expression, targeting the MAGI2/PTEN/PI3K axis [78]. Moreover, the primary transducer of the UPR, IRE1, disrupts de novo miRNA biosynthesis and maturation through the RIDD process. The RNase activity of IRE1 results in the degradation of tumor suppressor miRNAs such as miR-3607-3p, miR-374a-5p, and miR-96 in breast cancer cells. This pathway amplifies the expression of the miR-3607-3p target, the oncogenic factor RAB3B, establishing a positive association with cancer progression, invasion, and metastasis [79]. Liang et al. demonstrated that the hypoxia-induced ER stress/IRE1α/XBP1 pathway upregulates miR-153 expression in breast cell lines. miR-153 inhibits tumor angiogenesis by suppressing the HIF1α/VEGFA axis through binding to the 3′-UTR of HIF1A mRNA in breast cancer cells [80]. UPR-induced miR-616 is another miRNA that acts as a tumor suppressor in breast cancer by downregulation of c-MYC [81]. As mentioned above, the interaction between ER stress, ncRNAs, and the prognosis of breast cancer is complex and controversial. In certain pathways, the association between ER stress-related proteins and ncRNA expression aligns with the development of breast cancer, and other pathways following the suppression of tumor.

**Table 2:** Non-coding RNAs implicated in endoplasmic reticulum stress in breast cancer

Non-coding RNA	Mutual effects of non-coding RNA and endoplasmic reticulum stress in breast cancer	Study type	References
lncRNA MEG3	MEG3 overexpression increases the expression of endoplasmic reticulum stress-related proteins involved in the unfolded protein response, including GRP78, IRE1, PERK, and ATF6. It also upregulates proapoptotic proteins CHOP and caspase-3. Additionally, MEG3 overexpression enhances NF-κB expression, its translocation to the nucleus, and p53 expression	In vitro–In vivo	[7]
lncRNA MIAT	5-FU-induced endoplasmic reticulum stress increases the expression of GRP78 in MCF-7 cells. GRP78 could positively regulate the expression of MIAT and AKT through upregulating OCT4, thereby contributing to 5-FU resistance in BC cells. Additionally, the function of GRP78 silencing in promoting tumor cell sensitivity has been confirmed in vivo	In vitro–in vivo	[76]
lncRNAs CASC2, LINC00299, NEAT1	The association between the expression of lncRNAs CASC2, LINC00299, NEAT1, and the XBP1s/u ratio suggests that these lncRNAs have the potential to act as regulators of the unfolded protein response pathway in breast cancer	In vitro	[77]
miR-27a-3p	Endoplasmic reticulum stress promotes the secretion of exosomes, which contain elevated levels of miR-27a-3p. These exosomes are taken up by macrophages, leading to increased expression of miR-27a-3p and PD-L1 in macrophages. miR-27a-3p targets and negatively regulates MAGI2, while MAGI2 down-regulates PD-L1 by up-regulating PTEN, thereby inactivating the PI3K/AKT signaling pathway. The presence of exosomal miR-27a-3p reduces CD4+ and CD8+ T cells, IL-2 production, and promotes T cell apoptosis when macrophages and CD3+ T cells are co-cultured	In vitro	[78]
miR-3607, miR-374a, and miR-96	IRE1, a gene that frequently amplifies and overexpresses in aggressive luminal B breast cancer cells, is associated with worse overall survival. It mediates the degradation of tumor suppressor microRNAs including miR-3607, miR-374a, and miR-96 through Regulated IRE1-Dependent Decay (RIDD). Degradation of miR-3607 leads to elevated levels of RAB3B, a RAS oncogene GTPase, in breast cancer cells. Inhibiting IRE1 effectively suppresses proliferation and aggressive phenotypes in luminal breast cancer cells	In vitro	[79]
miR-153	Hypoxia-induced endoplasmic reticulum stress activates IRE1α and XBP1, leading to the upregulation of miR-153 expression. miR-153 is involved in fine-tuning the HIF1α/VEGFA axis in breast cancer angiogenesis. It directly binds to the promoter of the miR-153 host gene PTPRN, activating its transcription	In vitro–in vivo	[80]
miR-616-5p and miR-616-3p	In human breast cancer, the expression of miR-616 and its host gene CHOP is downregulated. During endoplasmic reticulum stress, both arms of miR-616 (miR-616-5p and miR-616-3p) are increased through the PERK pathway. Ectopic expression of miR-616 suppresses cell proliferation and colony formation, while knockout of miR-616 increases it. MiR-616 represses c-MYC expression by binding to its protein coding region. This repression of c-MYC by miR-616 leads to growth inhibition of cells	In vitro	[81]

## Therapeutic strategies based on non-coding RNAs and the ER stress in breast cancer

Current methods for managing breast cancer are diverse and include radiation therapy, cytotoxic-based chemotherapy, surgical mastectomy, hormone therapy, and treatment with monoclonal antibodies [82]. While chemotherapy drugs like carboplatin, paclitaxel, and doxorubicin have been discovered for breast cancer treatment, they come with limitations such as poor oral bioavailability, drug resistance, inefficient delivery, and high toxicity. Therefore, the development of anti-cancer drugs without side effects is crucial [83–85]. Researchers have shifted their focus to studying alternative biological targets and novel signaling pathways that can effectively inhibit breast cancer growth and metastasis [86]. Numerous studies have indicated that ncRNAs exhibit different expressions in various stages of development and pathological conditions, including breast cancer [87–90]. This suggests that ncRNAs may serve as an alternative treatment for cancer prevention and management in the future.

### ncRNAs and drug resistance in breast cancer

Despite the availability of several treatment methodologies, overcoming treatment tolerance remains a significant challenge in improving the clinical outcome of breast cancer [91]. Drug resistance poses a major obstacle to chemotherapy-based systemic treatment in metastatic and advanced breast cancer, contributing to lower recurrence-free survival rates in breast cancer patients [92, 93]. Previous studies have highlighted the involvement of ncRNAs, mainly long lncRNAs and miRNAs, in drug resistance in breast cancer [94]. For instance, miR-451 and miR-326 regulate drug resistance by controlling the expression of multidrug resistance 1 (MDR1) [95]. Other studies have shown that altered expression of miR-21 and miR-298 is associated with chemoresistance in breast cancer cells, targeting PTEN and MDR1, respectively [96, 97]. Additionally, miR-221/222 is linked to increased resistance to tamoxifen by targeting p27Kip1 in breast cancer [98, 99]. Investigations into trastuzumab-resistant breast cancer cells revealed that miR-200c downregulation leads to enhanced epithelial-mesenchymal transition (EMT) and tumorigenesis [100]. Moreover, miR-218 induces cisplatin resistance by targeting BRCA1 in breast cancer patients [101]. Long-term exposure to doxorubicin upregulates the intronic lncRNA Adriamycin Resistance-Associated (ARA) in doxorubicin-resistant MCF7 cells, contributing to inhibition of proliferation and increased drug sensitivity upon ARA deletion [102]. H19, a lncRNA, counteracts the downregulation of estrogen receptor α (ERα) protein induced by endocrine therapy, promoting treatment resistance in breast cancer cells [103]. Additionally, hypoxia-driven modulation of tumor suppressor ncRNAs and upregulation of carcinogenic ncRNAs play a pivotal role in creating resistance to various therapeutic agents in breast cancer cells. For instance, hypoxia inhibits miR-873-5p, increasing the expression of drug resistance-related targets such as MDR1 and pregnane X receptor (PXR) [104]. Hypoxia-induced inhibition of miR-326 expression, in a HIF1α-dependent manner, increases ITGA5 expression, resulting in chemotherapy resistance in triple-negative breast cancer (TNBC) cells [105]. Furthermore, hypoxia-induced miR-424 targets the apoptosis-related tumor suppressor PDCD4, conferring resistance to chemotherapies like doxorubicin and etoposide [106]. These examples illustrate the intricate involvement of ncRNAs in treatment resistance in breast cancer. Researchers continue to explore the molecular mechanisms of ncRNAs, their potential as biomarkers for predicting response, and as targets for overcoming drug resistance. Staying updated with the latest scientific findings is crucial for a comprehensive understanding of this complex issue as the field of ncRNA research evolves.

### ncRNAs as possible biomarker candidates for diagnosis and prognosis in breast cancer

The utilization of ncRNAs as therapeutic agents can be characterized by two criteria: (i) dysregulation of ncRNA in cancer cells compared to normal cells and (ii) alteration in the phenotype of cancer cells through the targeting of ncRNA expression [107, 108]. Reports indicate that miRNAs in serum can serve as diagnostic markers in patients. For instance, the down-regulation of let-7 family miRNAs is associated with a poor prognosis [107]. High levels of the miR-106b-25 cluster are significantly associated with shorter time to recurrence in breast cancer [109]. miR-181a and miR-221/miR-222 clusters are directly related to tumor progression and have diagnostic and prognostic value [110, 111]. Furthermore, reduced levels of miR-1247-5p are associated with age, tumor size, and a poor disease prognosis. Frères et al. demonstrated that the levels of miR-34a and miR-122 in both plasma and tumor tissue of breast cancer patients increased following neoadjuvant chemotherapy (NAC), particularly after anthracycline treatment. Subsequently, they employed a diagnostic test based on eight circulating miRNAs, namely let-7d, miR-103, miR-16, let-7i, miR-19b, miR-107, miR-148a, and miR-22, as an alternative to mammography for the diagnosis of breast cancer [112]. On the other hand, circRNAs, being stable biomarkers with high stability in serum, saliva, urine, milk, and exosomes, exhibit great potential for cancer diagnosis and monitoring disease progression. However, studies investigating their role in breast cancer are currently very limited [82, 113–115]. Analysis of The Cancer Genome Atlas (TCGA) data on 1097 breast cancer patients revealed significant differential expression of lncRNAs in various subtypes of breast cancer. In particular, 1510 lncRNAs were differentially expressed in normal versus TNBC samples, while 672 lncRNAs showed differential expression in non-TNBC versus TNBC samples. Among these, three upregulated lncRNAs (AP000924.1, AC091043.1, and FOXCUT) demonstrated promising potential as biomarkers for the diagnosis of TNBC. On the other hand, three other lncRNAs (AL354793.1, AC010343.3, and FGF10-AS1) were associated with prognosis in breast cancer patients. These findings highlight the relevance of lncRNAs in breast cancer and their potential utility as diagnostic and prognostic markers in TNBC [116]. But beyond the role of ncRNAs as a biomarker, ncRNAs have emerged as promising therapeutic agents in cancer treatment, with ongoing clinical trials demonstrating their diverse applications. miRNAs and small interfering RNAs (siRNAs) have garnered particular attention for their ability to precisely regulate gene expression, offering targeted therapeutic opportunities. Clinical trials investigating miRNA-based therapies have shown their value as diagnostic and prognostic markers, with some yielding promising results in intervention studies (ClinicalTrials.gov identifier: NCT05477667, NCT02247453, NCT03776630, NCT05346757). For example, the therapeutic efficacy of miR-34a has been demonstrated in advanced solid tumors, although challenges have been encountered, highlighting the importance of understanding potential adverse events. Furthermore, the recent approval of siRNA-based drugs by the FDA for non-cancerous diseases represents a significant advancement, opening doors for progress in cancer therapy [117]. Collectively, these investigations underscore the evolving landscape of ncRNA-based therapies, showcasing their potential across various cancer types and positioning them as promising candidates for future cancer management strategies.

### Novel therapeutic strategies based on ncRNAs and UPR in breast cancer

As previously mentioned, ER stress influences the expression of ncRNAs, and together, they synergistically impact the development of breast cancer. Within the realm of ncRNAs, miRNAs assume a crucial role in regulating the drug sensitivity of breast cancer cells by modulating the UPR signaling pathways [11]. Studies have indicated that ER stress can be regarded as a side effect induced by anticancer drugs like cisplatin, etoposide, doxorubicin, gemcitabine, cytarabine, and vinorelbine [118]. On the other hand, hypoxia created in tumor conditions can not only act as a factor to activate ER stress [37] but also act as an inducer of EMT in cancer [119, 120]. Furthermore, hypoxia may regulate the expression of various ncRNAs in both HIF signaling-dependent and independent ways [120]. Given the role of hypoxia in cancer progression in solid tumors, such as breast cancer, directly targeting HIFs provides an excellent therapeutic option in clinical settings [105, 121]. Numerous HIF-targeting inhibitors are currently undergoing preclinical trials for validation, either as standalone treatments or in combination with other regimens, to combat various advanced cancers. The production of HIF-1alpha in the tumor microenvironment induces the upregulation of PD-L1 in both myeloid-derived suppressor cells and tumor cells. Consequently, this process can suppress immune system responses against the tumor, contributing to immune evasion [122]. Yang et al. demonstrated the potential involvement of HIF-1a and miR-210 signaling pathways in breast cancer invasion and metastasis, suggesting that targeting these pathways could represent a novel therapeutic strategy for breast cancer [123]. Glycolysis has emerged as a primary metabolic process supporting anabolic growth and energy production in cancer cells. In breast cancer cells, inhibiting glycolysis is being explored as a novel therapeutic approach to address hypoxia and drug resistance associated with mitochondrial respiratory defects [124, 125]. Among the methods of treating tumor cells, chemotherapy usually fails because tumor cells acquire multi-drug resistance and lead to various outcomes, including ER stress tolerance (ERST) [126–128]. In addition, ER stress induces resistance to tyrosine kinase inhibitors by upregulating Bcl-xL [129]. Overall, UPR activation has been demonstrated to contribute to drug resistance. Hence, the exploration of small molecule inhibitors targeting UPR components holds promise as a strategy to overcome ER stress-induced drug resistance and sensitize cancer cells to apoptosis [130]. The overexpression of XBP1 has been observed in various human cancers, including breast cancer. Targeting XBP1, a crucial component of the UPR and a significant nuclear transcription factor, is being considered as a therapeutic strategy [131]. It is important to acknowledge that the diverse functions of the UPR and the targeting of this pathway in clinical settings can potentially lead to unintended side effects. Therefore, additional studies are necessary to thoroughly investigate and assess the possibility of unwanted side effects when specifically targeting the UPR. These studies are crucial for ensuring the safety and efficacy of UPR-targeted approaches in therapeutic interventions.

## Conclusion

The exploration of ncRNAs in recent years has illuminated their pivotal role as potential therapeutics in the management and progression of breast cancer. Current evidence underscores the initiation of the UPR by tumor cells in response to environmental changes, portraying the UPR as a pro-oncogenic mechanism influencing diverse aspects of breast cancer. This intricate relationship orchestrates the regulation of ncRNA levels in tumors, suggesting the potential use of UPR modulators as biomarkers in cancer drug therapy and prognosis. However, the downstream components of the UPR, under specific circumstances, exhibit a dual role—they not only regulate ER stress-induced apoptosis but also foster breast cancer proliferation by modulating ncRNA expression. Conversely, ncRNAs reciprocally influence the expression of downstream UPR target genes. In this review, we discussed the mutual regulation of ER and ncRNAs, emphasizing the need for further research, particularly in clinical studies, to elucidate the UPR’s role in tumorigenic mechanisms. While the promise of ncRNA-based therapies in cancer management is evident, it is essential to recognize associated limitations. Challenges include efficient delivery to target tissues and cells, necessitating ongoing investigation into safety and effectiveness. Precision in targeting specific ncRNAs within complex regulatory networks poses a significant challenge, requiring meticulous modulation to avoid disrupting normal cellular functions. Potential unintended consequences, such as off-target effects and interference with unintended pathways, warrant thorough preclinical validation. Moreover, addressing the dynamic nature of RNA molecules, including stability and pharmacokinetics, remains a challenge, necessitating strategies to enhance their presence and stability in targeted tissues. Despite these challenges, the ongoing efforts in the field of ncRNA-based therapies hold substantial promise for advancing breast cancer management toward more effective and tailored interventions.

## Data Availability

The datasets used during the current study are available from the corresponding author upon reasonable request.

## Abbreviations

ATF4: Activating transcription factor 4
BMSCs: Bone marrow-derived mesenchymal stem cells
BRCA1: Breast cancer type 1 susceptibility protein
CALR: Calreticulin
CHOP: C/EBP homologous protein
circRNA: Circular RNA
c-MYC: Cellular myelocytomatosis oncogene
EMT: Epithelial–mesenchymal transition
ER: Endoplasmic reticulum
ERAD: ER-associated degradation
ERST: Endoplasmic reticulum stress tolerance
GRP78: Glucose-regulated protein 78
GTL2: Gene trap locus 2
HIF1α: Hypoxia-inducible factor 1 alpha
HOTAIR: HOX antisense intergenic RNA
ICD: Immunogenic cell death
IRE1α: Inositol-requiring enzyme 1 alpha
JNK: C-Jun N-terminal kinase
lncRNAs: Long non-coding RNAs
MAPK: Mitogen-activated protein kinase
MDR1: Multidrug resistance 1
MEG3: Maternally expressed gene 3
miRNAs: MicroRNAs
NAC: Neoadjuvant chemotherapy
ncRNAs: Non-coding RNAs
NEAT1: Nuclear-enriched abundant transcript 1
NORAD: Non-coding RNA activated by DNA damage
P53: Tumor protein 53
PDIA6: Protein disulfide isomerase family a member 6
PDIs: Protein disulfide isomerases
p-eIF2α: Phosphorylated eukaryotic translation initiation factor 2 alpha
PI3K: Phosphoinositide 3-kinase
PKR: Protein kinase R
PTEN: Phosphatase and tensin homolog
PXR: Pregnane X receptor
RIDD: Regulated IRE1-dependent decay
TNBC: Triple-negative breast cancer
TRAF2: Tumor necrosis factor receptor-associated factor 2
TRAIL-R1/DR4: Tumor necrosis factor-related apoptosis-inducing ligand receptor 1/death receptor 4
UPR: Unfolded protein response
VEGFA: Vascular endothelial growth factor A
XBP1: X-box binding protein 1
